# Analysis and Comparison of Friction Stir Welding and Laser Assisted Friction Stir Welding of Aluminum Alloy

**DOI:** 10.3390/ma6125923

**Published:** 2013-12-18

**Authors:** Sabina Luisa Campanelli, Giuseppe Casalino, Caterina Casavola, Vincenzo Moramarco

**Affiliations:** Dip. di Meccanica, Matematica e Management, Politecnico di Bari, Viale Japigia 182, 70126 Bari, Italy; E-Mails: campanel@poliba.it (S.L.C.); casavola@poliba.it (C.C.); v.moramarco@poliba.it (V.M.)

**Keywords:** friction stir welding, hybrid laser friction stir welding, welding speed, microstructure, micro-hardness, residual stress

## Abstract

Friction Stir Welding (FSW) is a solid-state joining process; *i.e.*, no melting occurs. The welding process is promoted by the rotation and translation of an axis-symmetric non-consumable tool along the weld centerline. Thus, the FSW process is performed at much lower temperatures than conventional fusion welding, nevertheless it has some disadvantages. Laser Assisted Friction Stir Welding (LAFSW) is a combination in which the FSW is the dominant welding process and the laser pre-heats the weld. In this work FSW and LAFSW tests were conducted on 6 mm thick 5754H111 aluminum alloy plates in butt joint configuration. LAFSW is studied firstly to demonstrate the weldability of aluminum alloy using that technique. Secondly, process parameters, such as laser power and temperature gradient are investigated in order to evaluate changes in microstructure, micro-hardness, residual stress, and tensile properties. Once the possibility to achieve sound weld using LAFSW is demonstrated, it will be possible to explore the benefits for tool wear, higher welding speeds, and lower clamping force.

## 1. Introduction

Friction Stir Welding (FSW) is a relatively new welding process, which was developed at The Welding Institute (TWI), Cambridge, United Kingdom, in 1991.

FSW offers significant benefits over conventional joining processes so it has attracted attention as a solid state bonding method with low heat input and has become widespread as a lap joint technique to weld together aluminum alloy sheets [1]. A rotating tool with a shoulder and terminating in a pin moves along the butting surfaces of two rigidly clamped plates placed on a backing plate. Heat generated by friction at the shoulder and to a lesser extent at the pin surface softens the material being welded. Severe plastic deformation and flow of this plasticized metal occurs as the tool is translated along the welding direction [2,3].

Extensive research has been carried out to improve the applications of this process. Researchers have explored different aspects of this process namely, tool design, microstructural and mechanical properties, residual stress, and mathematical modeling.

Fujii [4] studied the effect of tool design on mechanical properties and microstructure of friction stir welded aluminum alloys. Arora [5] determined the optimum tool pin geometry from its load bearing capacity for a given set of welding variables and tool and work-piece materials. “Pin” and pinless tool configurations were used for thin sheets in AZ31 magnesium alloy friction stir welding. A different metal flow was observed depending on the presence or absence of the pin [6].

An attempt has been made to study the effect of tool pin profiles and welding speed on the formation of friction stir processing zone in AA2219 aluminum alloy. The square pin profiled tool and 0.76 mm/s speed produced mechanically sound and metallurgical-defect-free welds [7].

As an alternative tool design there is a class of FSW tools called bobbin tools (sometimes referred to as self-reacting tools). The name refers to the shape of these tools, which consist of two shoulders connected by the tool pin. When using a bobbin type tool there is no need for a backing plate. Hilgert [8] studied thermal models for bobbin tool friction stir welding.

Cerri and Leo [9] proposed a heat treatment of 2 h at 535 °C to improve the weld ductility of medium strength aluminum alloy, while the tensile strengths remained comparable to those of as-friction stir welded samples.

Fratini and Pasta [10] studied the longitudinal residual stress distributions in friction stir welding for butt and skin–stringer geometries, including lap and T configurations. The findings suggested that FSW of complex skin–stringer geometries produces higher residual stresses than those of butt joints. Bastier [11] used a steady-state algorithm, based on an elasto-viscoplastic constitutive law, to estimate the residual state induced by the process.

FSW has several disadvantages. As it is a solid state process, a great amount of tool wear takes place during the plunging stage as the work piece material is cold at this time. Weld speeds in FSW are slower which can lead to time-consuming joining process. As higher weld forces are required during this process, equipment used for FSW is massive and expensive [12]. Moreover friction stir welding of high melting temperature materials, such as steel and stainless steel are known to have welding tool limitations. Therefore, the use of standard FSW machines runs into high capital cost requirements and relatively poor productivity.

Therefore, a new modification of friction stir welding process, called Laser Assisted Friction Stir Welding (LAFSW), has been developed. LAFSW is a combination of FSW and laser welding, with FSW being the dominant process and laser welding playing a supporting role with the aim of pre-heat the parts. The use of the laser beam introduces additional local heating, immediately ahead of the weld zone so that less mechanical energy, delivered through the tool, must be converted into heat. This reduces the tool forces, deflections in the machine and fixture, and may enable higher weld speeds. The Laser Assisted-Friction Stir Welding was investigated by Kohn to join AZ91D Mg alloy plates [12].

Thereafter, few works have been addressed to this subject, which make the LAFSW substantially poorly explored. Nevertheless, the expected benefit of using this hybrid technique is considerable and, in addition, other new challenging materials as titanium alloys are under study [13,14].

More studies paid attention to laser as a preheating source to enhancing materials flow during FSW. Chang [15] used a 2 kW pulsed Nd:YAG (neodymium-doped yttrium aluminum garnet) laser for the AA6061-T6 Al alloy and AZ31 Mg alloy dissimilar weld. The optimum hybrid welding condition suggested to apply 2 kW laser power and could improve the dissimilar joint performance significantly.

The objective to perform FSW butt joints of dissimilar steel and aluminum sheets in a thickness of about 1 mm was reached by preheating the steel blank with a laser beam [16].

A fiber laser-assisted friction stir welding system was designed by Casalino [17]. The system combined a conventional commercial friction machine and a fiber pumped laser system. The aim was to investigate the influence of the LAFSW on the weld quality of aluminum alloy.

In this paper the LAFSW was studied experimentally for the aluminum alloy weld. An Ytterbium fiber laser was coupled with a 4 kW power FSW machine.

FSW and LAFSW tests were conducted on 6 mm thick 5754 H111 aluminum alloy plates, in butt joint configuration, with constant tool rotation rate and with different feed rates in order to compare the two processes and to evaluate changes in microstructure, micro-hardness, residual stress, and tensile properties induced by laser pre-heating. The temperature fields during and after the welding process were measured using both infrared camera and thermocouples.

The differences in the effects of the two welding processes were analyzed and the advantages of the LAFSW one were highlighted. By this investigation, the LAFSW effectiveness at welding aluminum alloys was demonstrated. Once the effectiveness of LAFSW is demonstrated, it will be possible to study its efficiency by investigating the effects on tool wear and process productivity.

## 2. Experimental Tests and Finite Element Analysis

**Figure 1:**
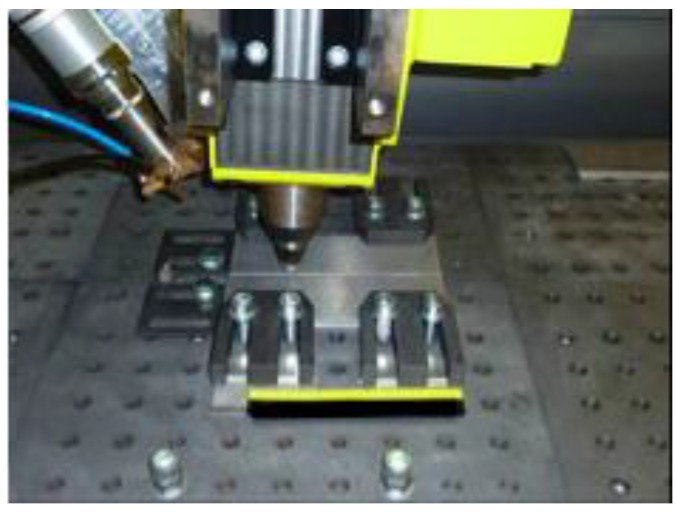
Machine set-up and clamping system.

**Figure 2:**
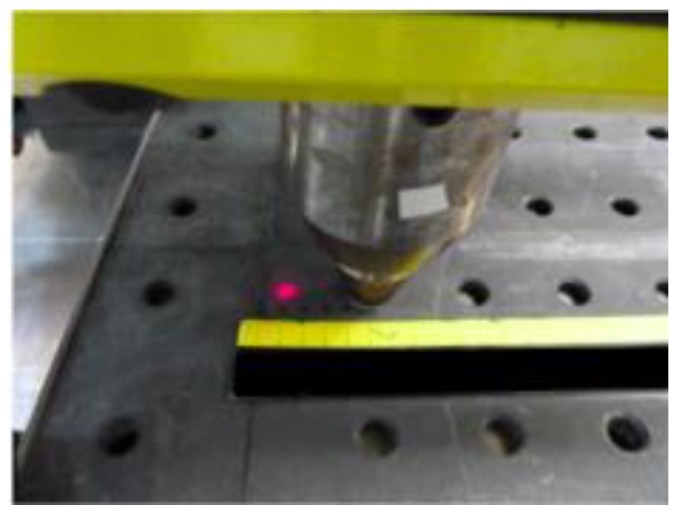
Distance between the laser spot and the pin tool.

The FSW machine was equipped with a Ytterbium fiber laser source, having a maximum power of 4 kW and a wavelength of 1070 nm. This laser source was used for preheating the aluminium plates in the hybrid welding process. The diameter of the focused laser beam was 1.2 mm, but in order to get available a larger area interested by the heating the beam was defocused to the maximum possible value. The laser was positioned aligned with FSW pin in order to generate heating along the welding direction (Figure 1). Moreover, the laser spot was located 40 mm forward of the tool, as illustrated in Figure 2.

The material under investigation was the 5754 H111 commercial aluminium alloy. The 5754 aluminium alloy is a non-heat treatable wrought alloy that can only be strengthened by cold deformation. Typical applications of this alloy are welded structures in nuclear, chemical, and food industries, marine and offshore applications, vehicle bodywork, and pressure vessels. The base material was supplied as 6 mm thick rolled sheet in H111 condition.

Rectangular plates were welded perpendicular to the rolling direction.

The welding process was carried out rotating the tool at 500 rpm, with a constant transverse speed of 20 mm/min, with a 2° tilt angle, and varying the laser power in HLFSW tests on three levels (500, 1000, and 2000 W), according to Table 1.

**Table 1:** Process parameters.

Designation	Traverse speed (mm/min)	Tool rotation speed (rpm)	Dwell time (s)	Laser power (W)
1	20	500	3	–
1L	20	500	3	500
2L	20	500	3	1000
3L	20	500	3	2000

### 2.1. Welds Shape and Microstructure Analysis

**Figure 3:**
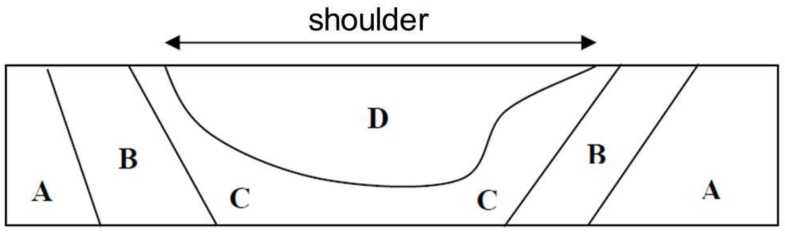
Typical butt joint profile.

The generalized profile of a butt joint, as proposed by TWI, is an inverted trapezoid displaying four zones (Figure 3). The first (A) is the unaffected base metal (BM), where no microstructural or property changes took place. The second (B) is the heat-affected zone (HAZ), where the material experienced no plastic deformation but was influenced by the heat of welding, leading to some microstructural changes. The third (C + D) is the thermo-mechanically affected zone (TMAZ), where the material has deformed and was also influenced by heat. Fourth (D) is the nugget, which is the recrystallized region of the TMAZ.

All the joints were cross-sectioned perpendicularly to the welding direction for metallographic analysis. The cross-sections of the metallographic specimens were prepared by standard metallographic techniques, etched by HF’s reagent (5 mL HF, 120 mL H_2_O).The microstructural behaviour of 5754 aluminium alloy joined by FSW and HLFSW was studied by employing an optical microscope in all the conditions of welding speed. Specifically, the microstructure was identified and the grain size was measured in all characteristic zones of FSW.

### 2.2. Temperature Measurement

The temperature field of the aluminium sheets during the welding process has been measured using a NEC H2640 infrared camera (range: 0–2000 °C; resolution: 0.06 °C; accuracy ±2 °C or ±2%; spectral range: 8–13 μm). The camera has been positioned normal to the weld direction with an angle of 30° between the optical axe of the camera and the normal direction at the aluminium sheet. To increase the emissivity ε, the specimen has been painted with matte black acrylic spray paint (ε = 0.95). The central part of the specimen has not been painted to avoid inclusion into the welded joint.

To measure the temperature along the weld line, also bead on plate tests were conducted with the same condition used for the weld joints, on full painted specimens.

Due to the temperature range limitation of the infrared camera, and because a higher temperature has been estimated for the specimen type 3L (Table 1), in addition, a thermocouple system has been used to measure the temperature during the process.

### 2.3. Residual Stresses Measurement

Several techniques can be used to measure residual stresses in metal components [18,19,20,21,22], but just some of these techniques are both quantitative and non-destructive. X-ray diffraction is probably the most widespread technique to measure the residual stress on the surface because it allows mapping the stress field, point by point, quite rapidly, keeping unchanged, the characteristics of the component.

Before measuring the residual stress, each specimen was cleaned with an acrylic lacquer thinner to remove the paint, while no further mechanical treatment was conducted to not modify the stress field of the surfaces.

**Figure 4:**
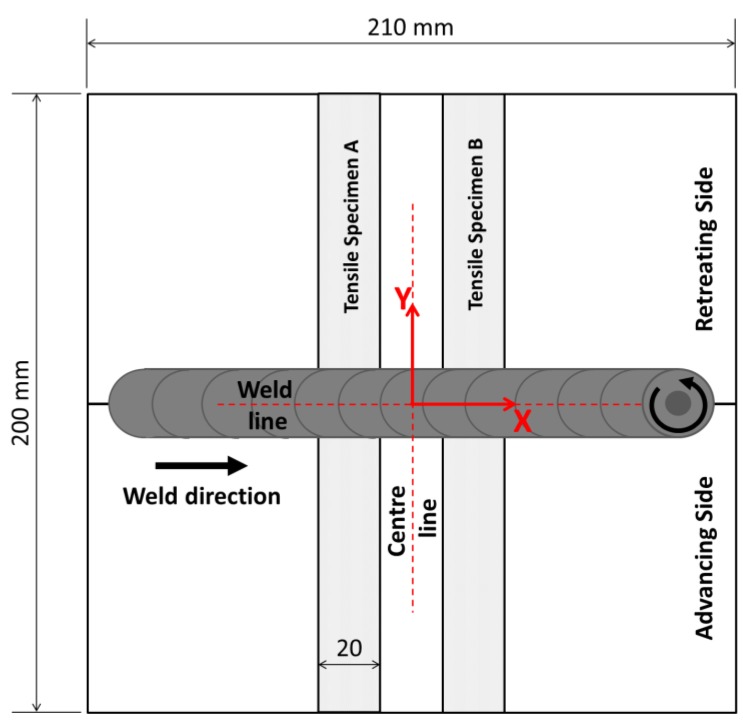
Schematic view of the weld: the tool transverse and rotation directions, the coordinate system and the measure lines are shown.

Analysis of the residual stresses has been performed using Xstress 3000 G3R Stresstech X-ray diffractometer (Stresstech Oy, Vaajakoski, Finland). It was instrumented with a Cr tube (λ = 0.2291 nm) and a 3 mm collimator. The X-ray voltage was set to 30 kV while the X-ray current was 8 mA. In each point the stress was measured at five different angles (0°, ±22.5°, ±45°) of which oscillation φ was ±3° and the exposition time was 40 s, which increased the deep penetration of the beam and avoided the shear effects. The detection distance was 75 mm. The residual stress measure was carried out along the center line (*x* = 0) and the welding line (*y* = 0) of the specimens (Figure 4). Both transverse (y-direction, $\sigma_{⊥}$) and longitudinal (x-direction, $\sigma_{∕∕}$) stresses have been measured for each point.

The X-ray diffraction theory is based on Bragg’s law:

*n*λ = 2*d*sinθ (1)

where *n* is an integer; λ is the wavelength of incident wave; *d* is the spacing between the planes in the atomic lattice; and θ is the angle between the incident ray and the scattering planes [23,24]. For a shear stress-free biaxial stress field in an un-textured material, the strain ε_ϕψ_ in the azimuthal direction ϕ and inclination ψ from the plane normal is determined by the equation:

(2) $$\varepsilon_{\varphi \psi }=\frac{d_{\varphi \psi }-d_{0}}{d_{0}}$$

where *d*_0_ is the unstrained inter-planar spacing. The use of the elasticity theory for an isotropic solid shows that the strain along an inclined line is:

(3) $$\varepsilon_{\varphi \psi }=\frac{1-\nu }{\text{E}}\left(\sigma_{1}\cos^{2}\varphi +\sigma_{2}\sin^{2}\varphi \right)\sin^{2}\psi -\frac{\nu }{\text{E}}(\sigma_{1}+\sigma_{2})$$

If we consider the strains in terms of inter-planar spacing, and use the strains to evaluate the stresses, then it can be shown that:

(4) $$\sigma_{\varphi }=\;\frac{\text{E}}{(1+\nu )\sin^{2}\psi }\left(\frac{d_{\psi }-d_{n}}{d_{n}}\right)$$

where *d*_n_ is the inter-planar spacing measured in normal direction to the plan. This equation allows calculating the stress in any chosen direction from the inter-planar spacing determined from two measurements, made in a plane normal to the surface and containing the direction of the stress to be measured. For stress determination the sin2ψ method was used. A number of XRD measurements are made at different ψ tilts, than the inter-planar spacing is measured and plotted as a curve. The stress can be calculated from the gradient of the line and with basic knowledge of the elastic properties of the material:

(5) $$\sigma_{\varphi }=\left(\frac{\text{E}}{1+\nu }\right)m$$

where *m* is the gradient of the *d vs.* sin2ψ curve.

### 2.4. Microhardness and Tensile Test

The Vickers microhardness of the weld zone was measured on all cross-sections perpendicularly to the welding direction and along three lines: at 1.5 mm from the root face using a Vickers indenter Remet HX 1000 50 gf load for 15 s.

Tensile specimens were prepared from material taken from the region indicated in Figure 4. Mechanical characterization under static load has been carried out on specimens 20 mm wide in order to evaluate the mechanical strength of welded components. Four electrical strain gauges (ER) have been bonded on both sides of each specimen in order to measure longitudinal strains during the tensile test and to evaluate secondary bending produced by occasional misalignment [25,26].

Tensile tests have been performed on an MTS Alliance RT/30 electromechanical machine equipped by a load cell capacity of 30 kN. The tests speed is 3 mm/min. Strain values have been recorded by means of System 5000 by Micro Measurement USA.

## 3. Results and Discussion

The following paragraphs summarize the experimental results of microstructure, micro-hardness, residual stress, and tensile test performed on specimens welded according to process parameters reported in Table 1.

### 3.1. Weld Bead Shape and Microstructure

**Figure 5:**
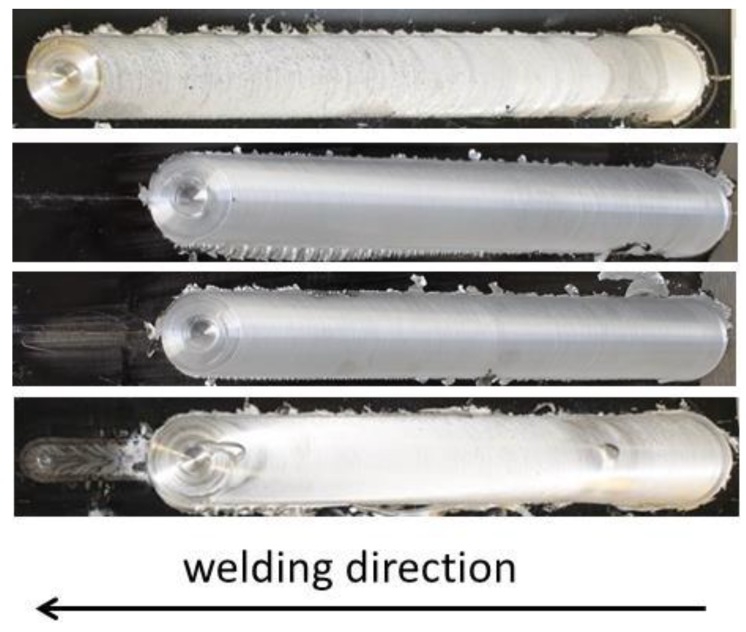
Outlook of weld surfaces.

Regardless of the range of welding conditions tested in this work, the welds obtained did not display any major structural defect. The weld crowns were regular in all produced welds. As shown in Figure 5, all the welds had a limited presence of flash and smooth and regular surfaces with regularly spaced semicircular bands. Sample 3L, which is the weld characterized by a laser power of 2 kW shows a clearly large fused zone on the surface of the weld, due to the high energy input given by the laser.

All the joints were cross-sectioned perpendicularly to the welding direction for metallographic analysis. Then they were polished, and etched. HF etching reagent (5 mL HF, 120 mL distilled water) was used to reveal the metallurgical structure.

**Figure 6:**
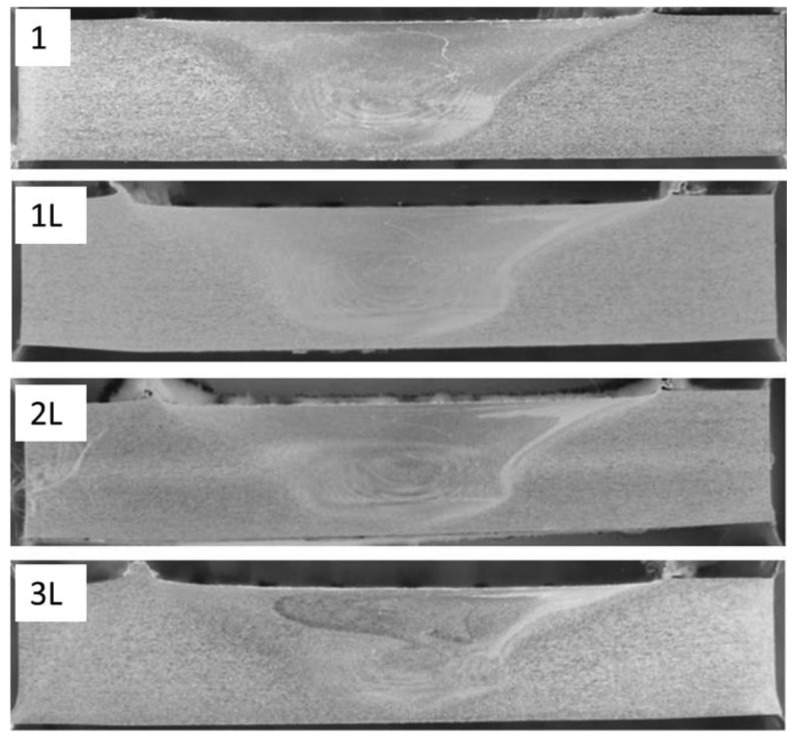
Macrographs of FSW and LAFSW showing the advancing side on the right and the retreating side on the left.

The analysis of FSW and LAFSW macrographs reveals characteristic features of FSWs in aluminium alloys, according to Figure 6.

**Figure 7:**
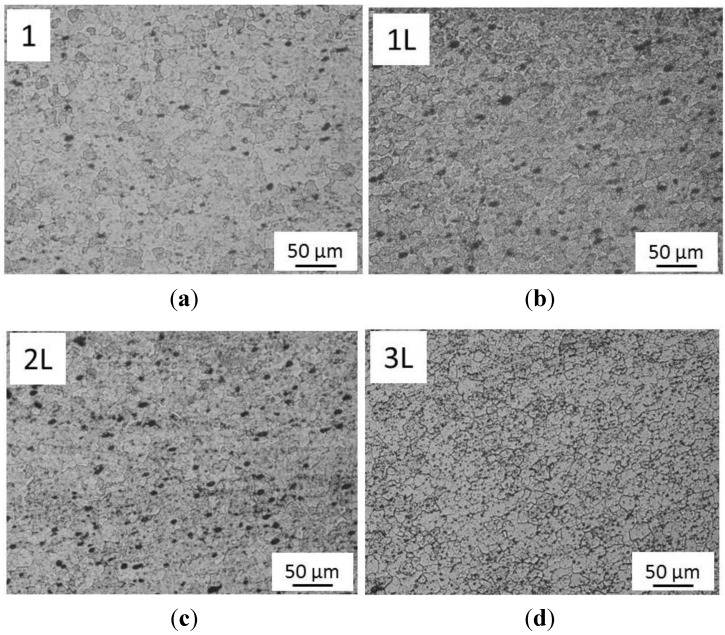
Nugget with its fine grains (200× magnification). (**a**) FSW weld; (**b**) LAFSW weld carried out with a laser power of 500 W; (**c**) LAFSW weld carried out with a laser power of 1000 W and (**d**) LAFSW weld carried out with a laser power of 2000 W.

**Figure 8:**
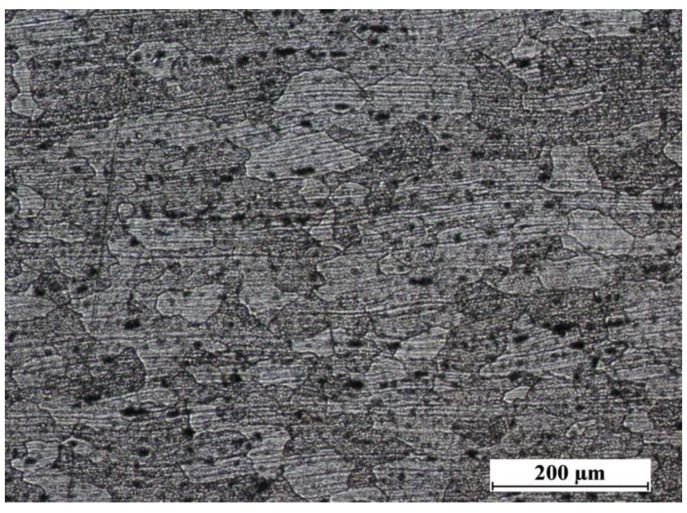
Microstructure of the base material (100× magnification).

**Figure 9:**
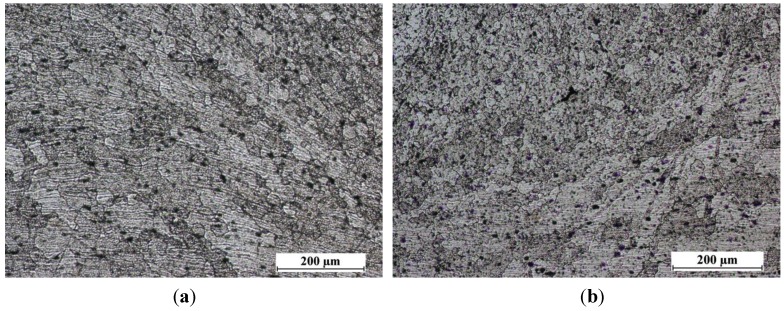
Transition zone in which HAZ and TMAZ are showed for (**a**) FSW; and (**b**) LAFSW welds (100× magnification).

The nugget is approximately symmetric about the weld centerline and it is typically similar in diameter to the pin. The microstructure within the weld nugget (Figure 7) consists of grain, which were much smaller, and equiaxed when compared to the parent metal microstructure (Figure 8). No difference was found in nugget’s structure of FSW and LAFSW welds (Figure 7), except for the LAFSW performed with a laser power of 2 kW for which the grain size appears smaller and with an equiaxic and regular shape just under the weld surface (Figure 7d). It can be explained with the fact that, for the 2 kW laser power, the weld line was fused just before the friction tool re-melted the material. The rapid solidification after the fusion brought to an initial smaller grain, which influenced the final size of the grain under the surface. In Figure 9a,b, the region adjacent to the TMAZ is the HAZ, displayed respectively for sample 1 and 3L. In this area the grain size is much larger than in the nugget zone and smaller than in the base metal.

### 3.2. Temperature Measurement

**Figure 10:**
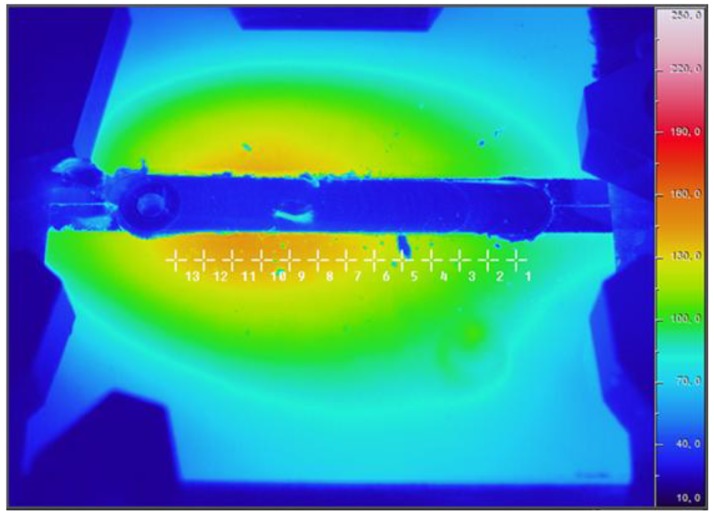
Example of infrared image. The marks indicate the points where temperatures are measured.

Temperature gradient reached during welding procedure and subsequent cooling, together with constraint geometry and other factors are responsible for the sign and level of residual stress generated into welded plates. Some authors, such as Hwang [27], and Zhu and Chao [28], have mapped the temperature field of the work piece during FSW process, but just a few works [29] have used thermography to analyze the temperature distribution during the process. In order to investigate on the relation between changes in laser beam power and the temperature distribution of the work piece, the maximal temperatures were recorded at ~15 mm from the weld line by means of a thermocamera (Figure 10).

**Figure 11:**
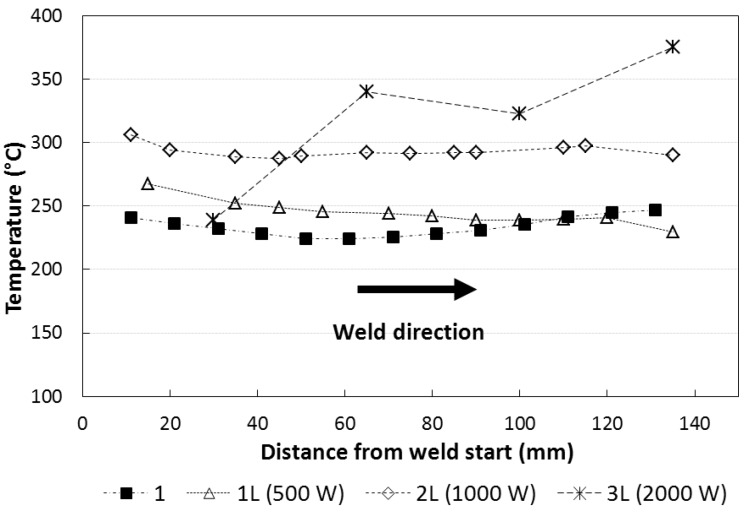
Maximal temperature at 15 mm from weld line during the welding process. (The 3L type specimen temperatures were measured using thermocouple while the others by infrared camera)

Figure 11 shows that the laser-beam produces an increase of the maximal temperature on the work piece. In the stationary zone of the process, from 40 mm to 120 mm from the weld start, the temperatures are: ~225 °C for type 1 junction; ~240 °C for type 1L junction; ~290 °C for type 2L junction, and ~330 °C for type 3L junction.

**Figure 12:**
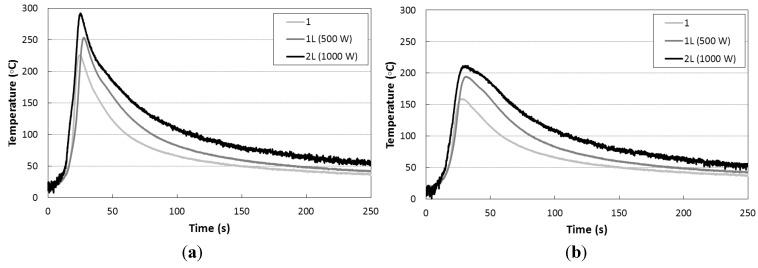

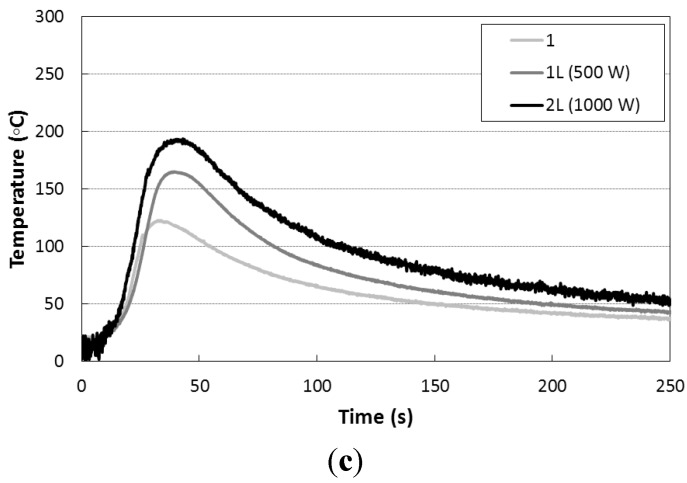
Temperature history during welding process at different distances from the cord. (**a**) Temperature *vs.* time at 1 cm from weld joint; (**b**) temperature *vs.* time at 2 cm from weld joint and (**c**) temperature *vs.* time at 3 cm from weld join.

The temperature history during welding process at different distances from the cord was pointed out in Figure 12. The results show that the maximal temperature at 2 cm from the weld cord is ~200 °C both for type 1L and 2L welds. Furthermore, it can be noted that the temperature of the work piece in the type 1L and 2L is quite constant after ~20 s after the onset of the peak of temperature at 1 cm, while for the type 1 the equilibrium occurs after ~35 s.

**Figure 13:**
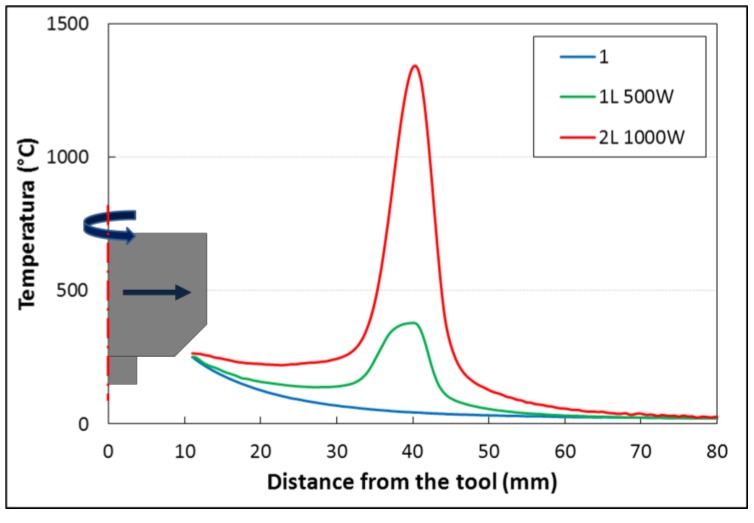
Comparison of temperature profiles along the weld junction between the friction stir welding and laser friction stir welding at 500 and 1000 W.

The infrared images of the welding process on the bead on plate specimens showed that both processes type 1L and type 2L show a “cold zone” between the laser spot and the tool. This effect is well pointed out in Figure 13, where the comparison of temperature profiles along the weld junction between the friction stir welding and laser friction stir welding at 500 W and 1000 W is shown. In both cases, the temperature measured between the laser spot and the tool is lower than the work temperature of the tool (260 °C).

### 3.3. Residual Stress Measurement

**Figure 14:**
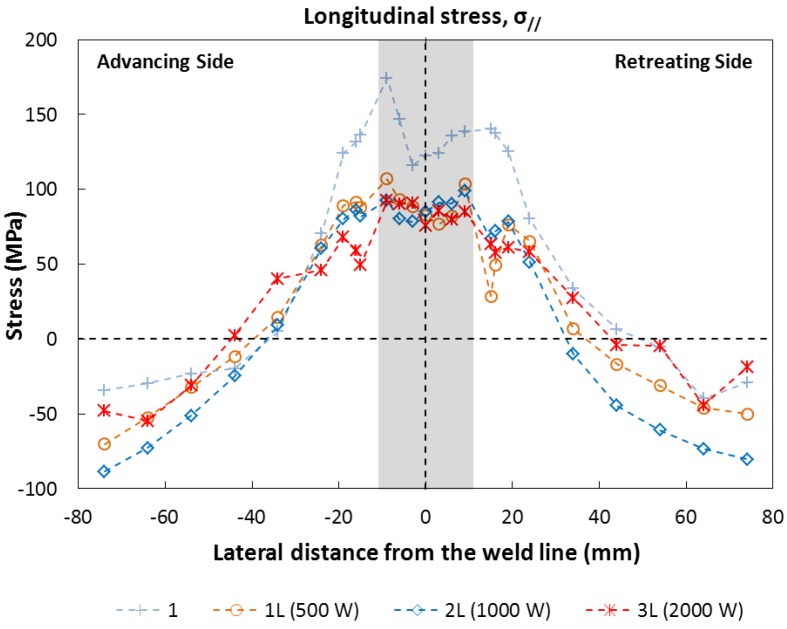
Longitudinal residual stresses along the centerline.

According to the geometry of the welding process, the largest residual stresses are expected parallel to the welding direction and close to the weld zone. The measured longitudinal residual stresses are shown in Figure 14. In all cases, the longitudinal stress field shows a tensile region near the cord and a compressive area at ~50 mm from the weld line. In particular, the type 1 specimen (friction only) shows the typical asymmetric stress field distribution near the cord [3]. Maximal stress tension was found in the advancing side and is ~150 MPa. In addition, in accordance with previous works [30] the results show a reduction of ~40 MPa of the residual stress in the central part of the cordon.

Generally, the hybrid laser beam friction stir welding plates show a reduction of the longitudinal residual stress close and into the cord in comparison with the normal friction stir welding, while no significant differences were found in the zone away from the weld line. The magnitude of this longitudinal tensile stress into the cord is ~90 MPa and it appears to be almost constant. Furthermore, this value seems to be independent from the increase of the power of the laser beam in specimen 1L, 2L and 3L. This effect could be explained by the fact that, although the laser power increases, the working temperatures near the tool remain the same as seen in Section 3.2.

**Figure 15:**
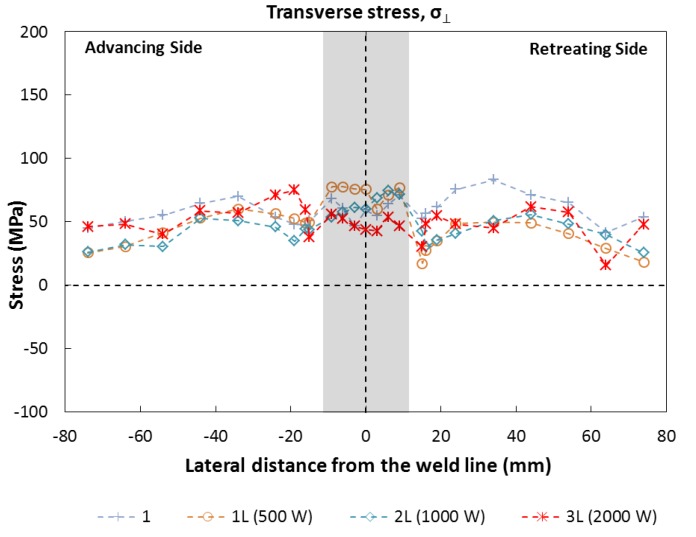
Transverse residual stresses along the centerline.

Figure 15 shows the transverse residual stresses within the weld as a function of distance from the weld line. In contrast with the longitudinal stresses, they do not display a direct dependence with the presence of the laser beam, but they indicate that the full specimen is under tension, due to the orientation of the lamination direction of the sheets.

### 3.4. Microhardness

**Figure 16:**
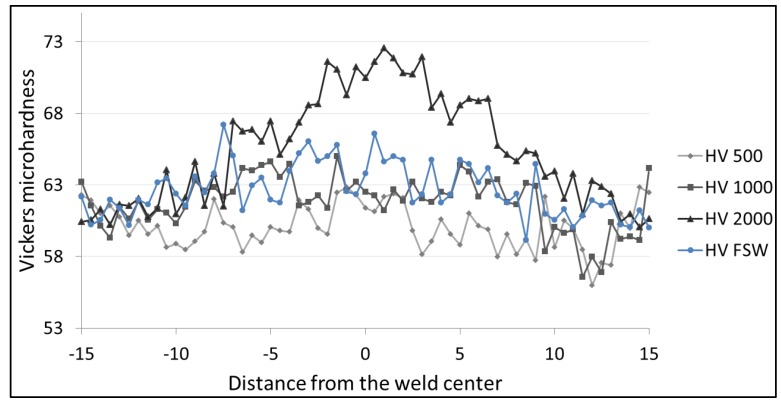
Microhardness profile across FSW and LFSW welds at 1.5 mm from the top surface.

**Figure 17:**
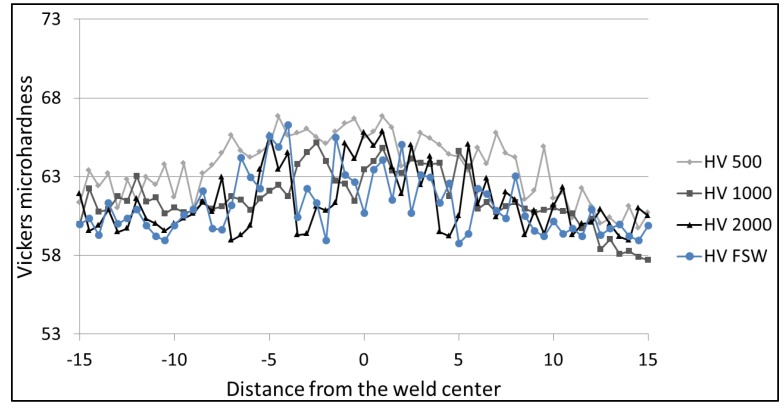
Microhardness profile across FSW and LFSW welds at the center.

**Figure 18:**
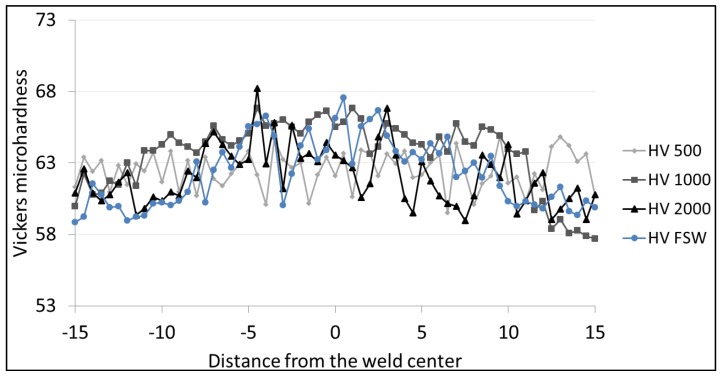
Microhardness profile across FSW and LFSW welds at 1.5 mm from the bottom surface.

Figure 16, Figure 17 and Figure 18 show the Vickers hardness distribution at the top (1.5 mm from the surface) at the center (3 mm from the surface) and at the bottom (1.5 mm from the back) of the weld. The base material hardness ranges between 60 and 65 HV. The observation of the hardness profiles shows that the LAFSW weld, with a laser power of 2 kW, highlights a slight increase in the weld nugget while all other profiles do not show significant changes from FSW to LAFSW, and between the different laser power levels.

No HAZ softening was found which was expected given the fact that tempered H111 is almost equivalent to the O-temper.

### 3.5. Tensile Test

Several workers have investigated the tensile properties of friction stir welds [30,31]. The studies of these mechanical properties are fundamental to understand the effect of laser on the friction stir welding process.

**Figure 19:**
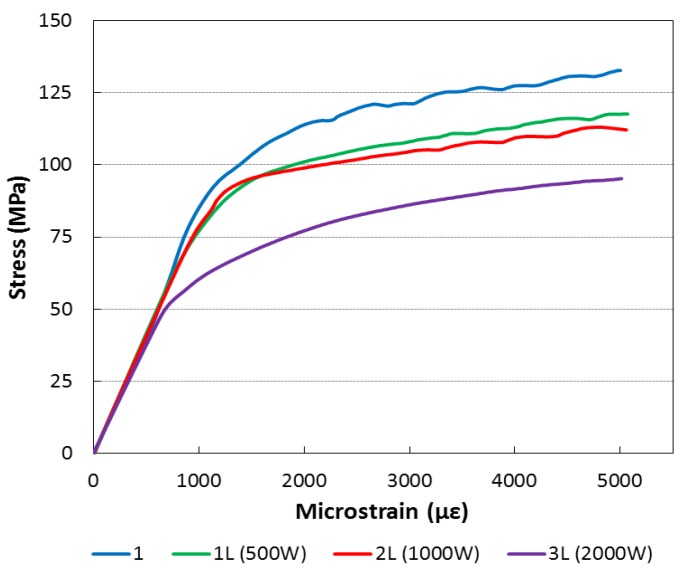
Elasto-plastic zone of tensile test (strain measured by strain gage).

**Figure 20:**
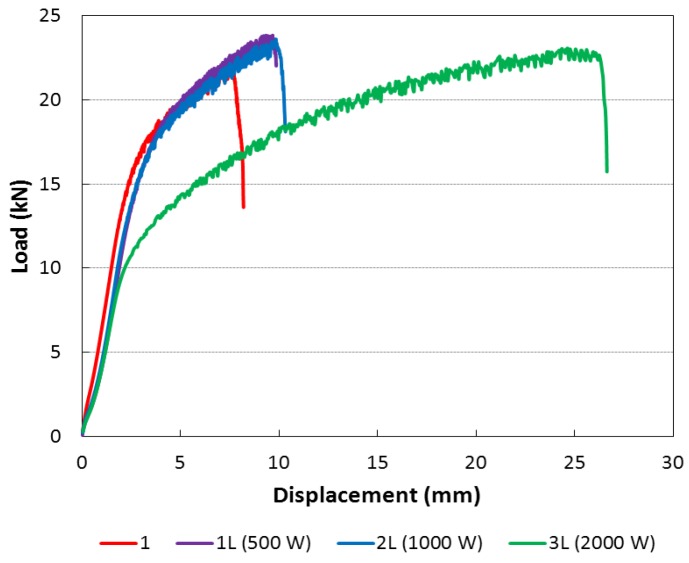
Complete tensile test.

Mechanical properties resulting from tensile tests are summarized in Table 2. The ultimate strength does not show significant variation between the specimens while the elongation at break increase with the power of the laser beam. Furthermore, the increase in the laser-beam power reduced the yield strength. Figure 19 shows the average stress/strain curves, measured experimentally by means of four strain gauges bonded on each type of welded specimens. The results allow underlining reduction of yield strength with the increase of the laser beam power. In Figure 20, the complete load-displacement curves, recorded directly from the testing machine, are reported. The results highlight the large increase in elongation at break for the 3L type specimens while the ultimate tensile strength is almost constant. The increase of the elongation at break and the reduction of yield strength for the 3L type specimen could be due to the higher level of heat input [30,31].

**Table 2:** Proof stress 0.2% and tensile strength of the specimens.

Designation	Proof stress 0.2% (MPa)	Tensile strength (MPa)	Elongation (%)
1	113.9	201.3	8.9
1L	101.0	207.3	9.4
2L	98.9	204.1	9.4
3L	77.2	206.9	14.8

**Figure 21:**
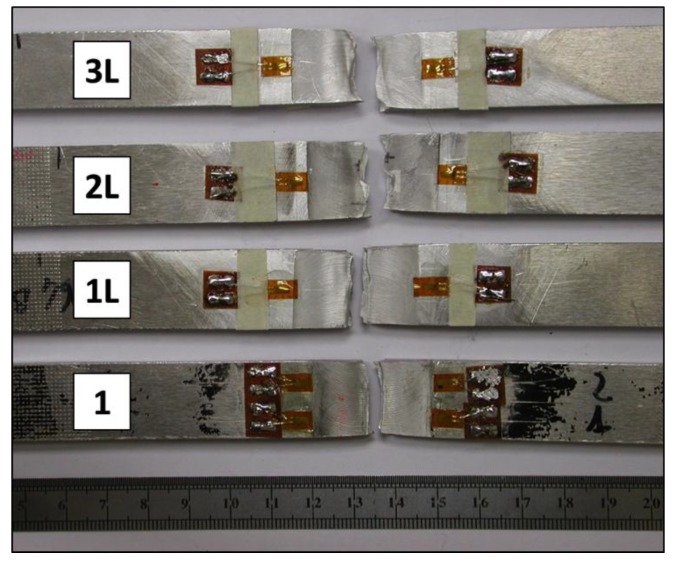
Specimens after tension test.

Figure 21 groups the specimens after the tension test. It can be observed that final fracture is perpendicular to the loading direction in the center of the welded joint. This effect could be due to the not complete penetration reached during welding process. All specimens show ductile fracture mode with cross-section shrinkage. The cracks starts from the reverse side of the specimen, inclined at approximately 45° to the tensile axis and grow in a straight line through the sample.

## 4. Conclusion

The effects of the Laser Assisted Friction Stir Welding on 5754 aluminium welding have been the subjects of this paper. In particular the changes in microstructure, micro-hardness, residual stress, and tensile properties induced by the laser pre-heating of the welding material were investigated.

It was found that the laser treatment induces higher microhardness values and lower longitudinal residual stress in the weld zone surface. The microstructures were not affected by change while the nugget shape was sensitively altered. The 2 kW laser pre-treatment produced a higher elongation, while the mechanical strength was unchanged.

Furthermore, the laser treatment seems to produce a substantial reduction in the magnitude of the transverse residual stresses that are a very critical issue in the classic friction stir welding process.

Moreover, the laser support allows preheating the plates and controlling the temperature gradient that usually is responsible for the thermal stress generation. It seems that, in the configuration adopted in this work, changes in laser power does not affect temperature gradient in the area around the tool and residual stress level keep constant.

It is possible to conclude that the laser pre-heating has a positive effect on the welding material. The flow material is facilitated around the tool pin while the surface hardness is improved at the same time.

As LAFSW of the aluminium alloy was successfully achieved, with some benefits for the weld, it will be reasonable to explore the benefits of this innovation for the tool wear, higher welding speeds, and lower clamping force.
